# Does Memory Accessibility Affect How Much We Learn from Studying?

**DOI:** 10.3390/bs15060760

**Published:** 2025-06-01

**Authors:** Nate Kornell

**Affiliations:** Department of Psychology, Williams College, 18 Hoxsey Street, Williamstown, MA 01267, USA; nkornell@gmail.com or nk2@williams.edu

**Keywords:** memory accessibility, learning, retrieval success, spacing

## Abstract

Two experiments were used to test the hypothesis that studying has a greater impact on learning when the information being studied is currently less accessible in memory. This hypothesis aligns with well-established findings like the spacing effect, but it is inconsistent with other evidence. Prior research has not directly tested the causal relationship between accessibility and learning. Two experiments were used to manipulate memory accessibility using semantic priming. The results indicated that differences in accessibility had no impact on learning. Retrieval difficulty and retrieval success also failed to influence learning. It is speculated that changes in accessibility that are specifically due to forgetting, rather than changes in accessibility per se, may have a causal impact on learning efficiency.

## 1. Introduction

Memories vary in how accessible they are. As a memory’s level of accessibility increases, the memory will tend to be retrieved with greater success and at faster speeds. Many factors affect memory accessibility; for the purposes of this article, the key ones are that semantic priming and studying both increase memory accessibility, while forgetting results in decreased memory accessibility.

Memory accessibility is correlated with learning: studying produces more learning when a memory’s accessibility is lower (e.g., R. A. Bjork & Allen, 1970; Cepeda et al., 2006; Cuddy & Jacoby, 1982). The main focus of this investigation was whether accessibility has a causal impact on learning. Based on previous research, it is possible that low accessibility causes increases in the amount that is learned by studying. It is also possible, however, that accessibility does not play a causal role; for example, in past studies, low accessibility has been coupled with more forgetting, and it is possible that forgetting, rather than accessibility, was playing a causal role. In the present study, the following question was asked: when forgetting does not vary between conditions, will differences in accessibility lead to differences in learning?

### 1.1. Evidence That Accessibility Is Correlated with Learning

Copious evidence has shown a relationship between learning and accessibility. The largest source of evidence is research on the spacing effect: Studies have shown that spacing study trials on the same item apart leads to more learning than massing them together (e.g., Cepeda et al., 2006; Rohrer et al., 2014). As the gap between two learning trials grows larger (i.e., the spacing increases), the accessibility of the memory during the second learning trial decreases. In other words, spacing decreases accessibility and increases learning efficiency (e.g., Rohrer, 2009; Rohrer & Taylor, 2006).

Other evidence, beyond spacing effects, has also shown a correlation between accessibility and learning. For example, R. A. Bjork and Allen’s (1970) participants studied twice and carried out a short activity between the study trials. The researchers found that when the activity caused more memory interference and, thus, more forgetting, the second study trial became more powerful. Cuddy and Jacoby (1982) also found that forgetting helped memory. Storm et al. (2008) found that learning increased when retrieval-induced forgetting caused the accessibility of memories to decrease.

### 1.2. Why the Hypothesis That Low Accessibility Causes More Learning Needs Further Testing

Although decreased accessibility and increased learning are correlated, they might not be related causally. Correlational evidence is always open to multiple explanations, and in this case, it is possible that a third variable (such as forgetting) played a causal role and accessibility did not. Moreover, past theorizing has explained the relationship between learning and accessibility without relying on accessibility as a causal factor. In the case of the spacing effect, many theories have been proposed to explain the value of spacing (e.g., Glenberg, 1979; Hintzman, 1974; Ross & Landauer, 1978), including stimulus sampling theories and study-phase retrieval theories, as described below. The present research was not designed to test these theories; rather, it was designed to investigate the possibility that variations in accessibility itself—which is a plausible explanation for the spacing effects given the correlation between accessibility and spacing—have a causal impact on learning.

One prominent class of theories is based on stimulus sampling theory (Estes, 1955; Madigan, 1969) and its application to spacing effects (e.g., Melton, 1970). One such theory, the New Theory of Disuse (R. A. Bjork & Bjork, 1992, 2006), claims that as accessibility decreases, there is an increase in the amount of long-term learning that results from studying. However, this theory does not assume that accessibility plays a causal role; it posits that fluctuations in learning conditions (e.g., contextual variability) that happen over time are responsible for the fact that learning increases as spacing increases.

A second class of theories posits that the spacing effect occurs because the second presentation of an item reminds participants of the first and induces a process of study-phase retrieval (Benjamin & Tullis, 2010; Hintzman, 2010; Hintzman et al., 1975; McKinley & Benjamin, 2020; Thios & D’Agostino, 1976; Wahlheim et al., 2014). Spacing makes the retrieval of a previous learning event more difficult (e.g., R. A. Bjork, 1988) and effortful (e.g., Pyc & Rawson, 2009). For example, Pavlik and Anderson (2005) proposed a theory in which lower levels of memory activation at the time of study are associated with more learning. Again, though, this theory does not assume a causal role for accessibility, but rather suggests that forgetting is a necessary ingredient to enhance learning.

To examine the causal relationship between decreased accessibility and increased learning, I conducted two studies that isolated the effects of accessibility by creating conditions that differed in accessibility but not forgetting. The manipulation involved conceptually priming some words (high accessibility) and not others (low accessibility) prior to restudy. Conceptual priming involves presenting a word that is related to the target word in order to increase the accessibility of the target word in memory (e.g., Collins & Loftus, 1975; Hutchison, 2003; Roediger & Challis, 1992; Soler et al., 2015). On primed trials, participants were shown sentences that contained two words that were related to the target word (for example, “Hawaii is a paradise on earth” was used to prime the word “island”). On unprimed trials, the sentence and target word were not related. If higher accessibility causes less learning to occur, then priming an item prior to restudy should increase accessibility, decrease learning during restudy, and thereby decrease performance on a final test.

### 1.3. The Importance of Difficulty for Learning

When a memory becomes less accessible, retrieving the memory becomes more difficult. According to the desirable difficulty framework (e.g., R. A. Bjork, 1994; E. L. Bjork & Bjork, 2011), difficulty during learning can make studying more effective and result in more long-term learning (Soderstrom & Bjork, 2015). Examples of desirable difficulties include spaced practice (Cepeda et al., 2008), interleaving (Rohrer et al., 2019), and testing oneself (Roediger & Butler, 2011). But not all forms of difficulty are desirable for learning–for example, multitasking increases difficulty and impairs learning (Sana et al., 2013). This research examines whether retrieval difficulty is a desirable difficulty.

Recent studies have examined the effect of retrieval success on learning. Retrieval success and accessibility are not the same thing, because retrieval success can be affected by factors other than accessibility, such providing hints or allowing more time for retrieval attempts. Accessibility and retrieval success are related, however, because memories with higher accessibility are more likely to be retrieved successfully. Research has shown that unsuccessful retrieval attempts enhance subsequent learning under some conditions (Grimaldi & Karpicke, 2012; Hays et al., 2013; Knight et al., 2012; Kornell et al., 2009; Richland et al., 2009; Yan et al., 2014). More importantly for the present study, successful and unsuccessful retrieval attempts do not seem to produce different amounts of learning (Chan et al., 2024; Kornell et al., 2015; Maddox et al., 2018; Vaughn et al., 2017; for a review see Kornell & Vaughn, 2016). For example, giving participants carefully designed hints while they carry out retrieval practice does lead to more retrieval success during learning, but it does not impact learning as measured on a subsequent final test (Vaughn & Kornell, 2019). If the present studies show that priming does impact retrieval success, but does not impact learning, then they would add to the accumulating evidence that retrieval success does not impact learning.

## 2. Experiment 1

Prior research has shown that lower accessibility is correlated with more learning. Experiment 1 investigated whether this correlation is based on a causal relationship whereby low accessibility causes an increase in the amount of learning that results from studying.

### 2.1. Method

Experiment 1 consisted of three phases. First, the participants studied a word list. Then, they restudied the words under two conditions: a given word was either primed and then restudied (high accessibility) or not primed and then restudied (low accessibility). Memory for the words was tested at the end of the experiment. Experiments 1 and 2 were both preregistered at https://osf.io/st4dx/ (accessed on 21 May 2025). The preregistration includes the methods, stopping rule for data collection, predictions, and data analysis plan.

#### 2.1.1. Participants

I conducted a power calculation assuming a within-participant *t*-test with alpha = 0.05, two tails, and a power of 0.90. I assumed an effect size of d = 0.30 because, in a novel paradigm like this, a small estimated effect size seems appropriate (e.g., d = 0.20), but the theory being tested is that accessibility causes the spacing effect, and spacing effects often have large effect sizes, so a slight upward adjustment of the estimated effect size seemed appropriate. The recommended number of participants was 119.

I preregistered a stopping rule for data collection. The stopping rule made data collection more efficient while also allowing for a larger sample to be run when appropriate, which helps account for uncertainties in the power analysis (see Dienes, 2016). I planned to recruit 100 participants. After collecting the data and excluding participants based on the criteria described below, I analyzed the data using a Bayes Factor analysis to compare the primed condition to the non-primed condition. I used the following stopping rule: if BF_10_ was less than 0.33 or greater than 3.00, I would stop data collection, but if it fell between these values, I would recruit another 50 participants and repeat this process until I had recruited a maximum of 200 participants.

I excluded participants, based on the preregistered analysis plan, for the following reasons: they did not finish the procedure (*n* = 3), they said they had participated in the study before (*n* = 6), the correlation between their pleasantness ratings in phase 1 and 2 was below 0.10 (*n* = 1; this analysis was meant to detect participants who did not pay attention while making the ratings), they said to exclude them because there had been technical problems (*n* = 2), their accuracy in judging whether sentences made sense was less than 0.70 (*n* = 6), or they went through the trials too quickly (*n* = 8). Going through the trials too quickly was defined as having a median response time under two seconds on pleasantness trials (collapsing across phase 1 and phase 2), having a median response time under five seconds on the sentence judgment task, and having a median response time under five seconds on the final test trials. This rule is a deviation from the preregistered exclusion rule, which said that participants would be excluded if any of these three criteria were met, rather than only being excluded if all three were met. However, the preregistered rule was a typing mistake, and the original intention was to exclude participants if all of these conditions were met, not if any were met. Thus, I deviated from the original rule (which would have meant excluding more than half of the participants) and used the intended rule.

Participants were recruited using Prolific and paid USD 3.00. After collecting the first batch of 100 participants’ data, the stopping rule was satisfied, so data collection ended.

The final sample included 77 participants. They were an average of 25 years old; 34 were female, 41 were male, 1 was non-binary/other gender, and 1 did not report their gender.

#### 2.1.2. Materials

The stimuli consisted of target words as well as sentences designed to prime them. To create the stimuli, I used the word-pair norms published by Nelson et al. (1998). I selected word pairs such that the forward association strength (i.e., the rate at which people said the target when given the cue) was between 0.2 and 0.5. Among these pairs, I selected pairs for which a given target had at least two cues in the stimulus set. If a target had more than two cues, I removed cues so that there were only two. I then filtered the stimuli so that all of the words (including targets and primes) were nouns. This filtering produced a set of target words that each had two words that could be used to prime them. For example, the word OCEAN was selected along with primes SEA and WAVE. These primes should elicit OCEAN because the forward association strength was 0.46 for SEA–OCEAN and 0.30 for WAVE–OCEAN.

From this stimulus set, I removed cases where a word (target or prime) was used more than once. If two targets were too similar, I removed one of them. I also removed targets if their two primes related to different two meanings of the target (clay–mold, mildew–mold).

Next, I created sentences that would serve as primes during the experiment. Each sentence contained both prime words for a given target. For example, for the target word OCEAN, which was primed by SEA and WAVE, one of the sentences was “The sea wave was huge”. I made two versions of each sentence; one version made sense and the other did not. In this example, the sentence that did not make sense was “The sea wave was tree”. The two sentences only varied by their last word, so that participants would have to read the whole sentence before deciding whether or not the sentence made sense. None of the sentences included any of the target words. In all, the stimuli consisted of 102 target words, each of which was accompanied by two sentences, one that made sense and one that did not.

Because I wanted to test participants by showing them the first two letters of each word, I divided the target words into two sets. One consisted of 64 words whose first two letters were unique within the set. These words were used as targets in the experiment. The remaining 38 words, which had the same first two letters as one of the other targets, were not used as targets, but their sentences were presented during the priming phase of the experiment as non-priming sentences.

#### 2.1.3. Procedure

**Figure 1:**
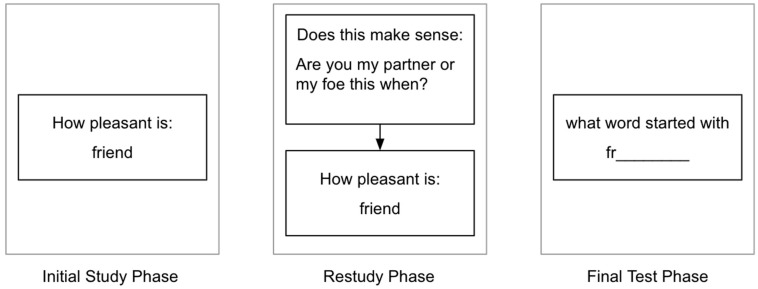
A sample trial from each of the three phases of Experiment 1. Note: During the restudy phase, some sentences made sense and some did not, and some sentences primed (i.e., were related to) the target word and others did not. In this example, the sentence did not make sense but it did prime the target word.

Following a demographics survey and initial instructions, the experiment was made up of three main phases (see Figure 1).

During phase 1, the initial study phase, participants made pleasantness ratings on a 1–5 scale for 48 target words. Each rating was made on a separate trial and participants were given unlimited time to make their ratings. These 48 target words were randomly selected from the 64 possible target words in the stimulus set, and then put in random order, uniquely for each participant.

During phase 2, the restudy phase, there were 48 trials. At the start of each trial, participants were shown a sentence and asked to choose between buttons labeled “makes sense” and “does not make sense”. There was no time constraint on responding. After they responded, during the second half of the trial, they were shown a target word and asked to make a pleasantness rating using the same procedure as in phase one.

Phase 2 is when the priming manipulation occurred. Half of the words were primed and half were not. For the 24 words that were primed, the sentence was related to the target word that followed it (e.g., “The sea wave was huge” would be followed by “ocean”). For the 24 words that were not primed, the sentence was not related to the target word (e.g., “Does the dough have butter in it?” would be followed by “school”). In addition, half of the sentences made sense in each condition and half did not. The order in which the words were presented was randomized anew for each participant, with the constraint that words presented in the first half of phase 1 were always presented in the first half of phase 2.

Phase 3, the final test phase, began immediately after phase 2 ended. The participants were tested on all 48 words. On each test trial, the participants were shown the first two letters of a target word and asked to type in the word. There was no time limit on responding. The order of the words was again randomized with the constraint that words presented in the first half of the other two phases were also presented in the first half of this phase.

After the final test phase, the participants were asked whether their data should be excluded because of problems during the study and whether they had taken part in the experiment before.

### 2.2. Results

**Figure 2:**
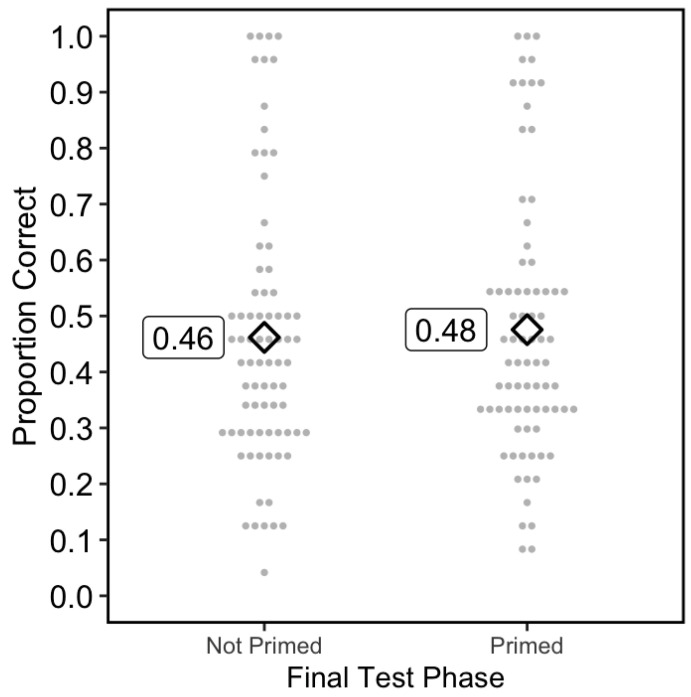
Results of Experiment 1. Note. The diamonds represent the overall means and each circle represents the mean of an individual participant. Each participant was in both conditions (i.e., is represented by two points, one on the left and one on the right).

On the final test, the accuracy in the Primed and Not Primed conditions was 0.476 (SD = 0.238) and 0.462 (SD = 0.240), respectively (see Figure 2). A Shapiro–Wilk test showed a violation of normality, W = 0.966, *p* = 0.036. A Bayesian Wilcoxon signed rank test showed that BF_10_ = 0.243. Because the initial hypothesis was directional, with the prediction that non-primed trials should lead to more learning than primed trials, I conducted a version of the Bayesian Wilcoxon test with the alternative hypothesis of not-primed > primed, and found BF_10_ = 0.060. These results support the null hypothesis that accessibility did not impact learning.

For completeness, I also carried out a 2 × 2 ANOVA on the final test accuracy, examining two variables: priming and sentence type (i.e., whether sentences made sense or not). The main effects and interaction were not significant. There were no significant main effects of priming (*F*(1, 76) = 1.07, *p* = 0.30, BF_incl_ = 0.18) or whether or not the sentences made sense (*F*(1, 76) = 0.04, *p* = 0.84, BF_incl_ = 0.14), nor was there a significant interaction (*F*(1, 76) = 3.83, *p* = 0.05, BF_incl_ = 0.16). (For more information about the BFincl statistic, see van den Bergh et al., 2020).

When the participants judged whether or not the sentences made sense, they were correct on 0.88 and 0.91 of trials in the Does Not Make Sense and Makes Sense conditions, respectively. Finally, I examined the pleasantness ratings during the restudy phase. Priming did not affect feelings of pleasantness. The average pleasantness ratings were 3.298 versus 3.298 for Primed and Not Primed (BF_10_ = 0.126).

### 2.3. Discussion

The results did not support the theory that accessibility is causally linked to learning. Priming a word, which presumably increased its accessibility on the subsequent study trial, did not affect performance on the final test.

Experiment 1 did not measure accessibility during the restudy phase, leaving open the possibility that the sentence primes did not actually influence accessibility during the restudy phase. Experiment 2 was designed to replicate Experiment 1, but also verify that priming impacted accessibility by having participants retrieve the target words from memory during the restudy phase.

## 3. Experiment 2

The only meaningful difference between Experiment 1 and 2 was that in Experiment 2, during the restudy phase, priming was followed by a retrieval attempt coupled with feedback, rather than a pleasantness trial. The retrieval attempt made it possible to measure memory accessibility during phase 2, and the feedback ensured that participants had a learning opportunity during every trial.

### 3.1. Method

#### 3.1.1. Participants

Participants were excluded, based on the preregistered analysis plan, for the following reasons: they did not finish the whole study (*n* = 13), they said they had participated in the study before (*n* = 9), they said to exclude them because there had been technical problems (*n* = 6), their accuracy in judging whether sentences made sense was less than 0.75 (*n* = 29), their median response time on pleasantness trials was less than 1.5 s (*n* = 9), or their median response time on test trials was less than 3.5 s (*n* = 2).

The participants were recruited using Prolific and paid USD 3.00. I used the same preregistered stopping rule for data collection as Experiment 1. Following the stopping rule, I collected data from a batch of 100 participants, and then two batches of 50 participants each. The final sample included 145 participants. They were an average of 30 years old; 87 were female, 55 were male, and three were non-binary/other gender.

#### 3.1.2. Procedure

**Figure 3:**
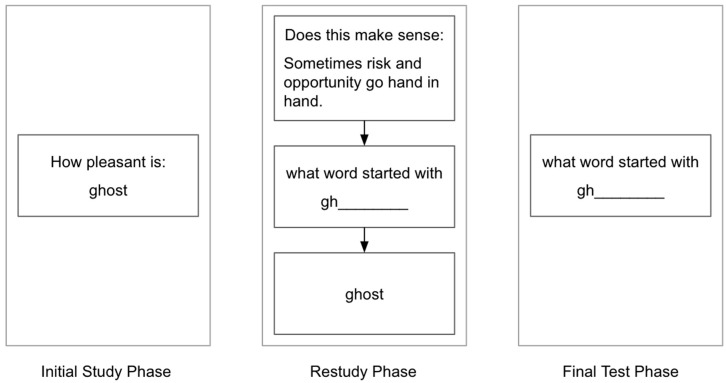
A sample trial from each of the three phases of Experiment 2. Note: During the restudy phase, some sentences made sense and some did not, and some sentences primed (i.e., were related to) the target word and others did not. In this example, the sentence did make sense but it did not prime the target word.

The materials were the same as in Experiment 1. The main procedural change occurred during the restudy phase (see Figure 3). As in Experiment 1, each restudy trial began with a sentence that either did or did not prime the word that followed. In the second half of each trial, instead of restudying via pleasantness trials, the participants were given a two-letter stem (the same stem they would see during the final test) and asked to recall the target word from the initial study phase. After they typed in their answer and pressed enter, they were shown the correct answer for two seconds.

The only other procedural change was that at the end of the study, the participants were asked whether they noticed that the sentences and the word that came after them were sometimes related.

### 3.2. Results

**Figure 4:**
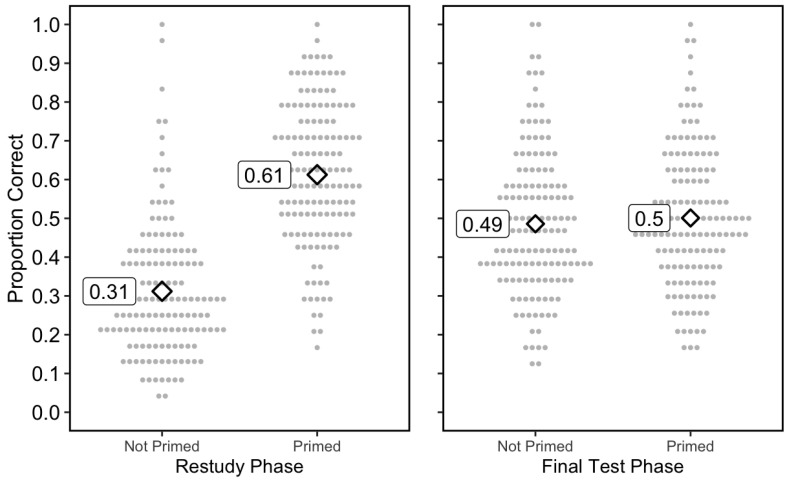
Results of Experiment 2. Note: The diamonds represent the overall means and each circle represents the mean of an individual participant. Each participant was in both conditions in each of the panels (i.e., is represented by two points in each panel, one on the left and one on the right).

I first analyzed performance on the test during phase 2 (see Figure 4, left panel). There was a significant effect of priming (BF10 = 5.20 × 1037, d = 1.58). The participants recalled almost twice as many words in the Primed condition (*M* = 0.613, SD = 0.182) as the Not Primed condition (*M* = 0.312, SD = 0.174).

During the final test, there was no effect of priming (BF_10_ = 0.22, d = 0.11) (see Figure 4, right panel). The mean proportions correct were 0.501 (SD = 0.176) and 0.486 (SD = 0.185) for the Primed and Not Primed conditions, respectively. Moreover, because the hypothesis was directional, namely, that performance would be higher in the Not Primed condition than the Primed condition, I computed a Bayes Factor using an alternative hypothesis of Not Primed > Primed, and found BF_10_ = 0.041. (Note that unlike Experiment 1, normality was not violated—Shapiro–Wilk W = 0.984, *p* = 0.080.)

Whether or not the sentences made sense did not affect the accuracy during restudy (BF_10_ = 0.247) or the final test (BF_10_ = 0.097). I also conducted a 2 × 2 ANOVA on final test accuracy, examining two variables: priming and sentence type (i.e., whether sentences made sense or not). The main effects and interaction were not significant. There were no significant main effects of priming (*F*(1, 144) = 1.76, *p* = 0.19, BF_incl_ = 0.15) or whether or not the sentences made sense (*F*(1, 144) = 0.08, *p* = 0.77, BF_incl_ = 0.08), nor was there a significant interaction (*F*(1, 144) = 0.001, *p* = 0.97, BF_incl_ = 0.01).

For completeness, I performed a conditional analysis comparing the final test performance for items that participants had answered correctly during phase 2 (*M* = 0.68) and items they had not answered correctly (*M* = 0.33). This difference is likely due to an item-selection effect; participants were more likely to think of an answer on the final test if they had thought of the same answer during the restudy phase.

On the final question, which asked how often the sentence and word were related during the study phase, 78% of participants said about half the time (which is correct), 14% said almost always, and 8% said almost never.

### 3.3. Discussion

Experiment 2 replicated Experiment 1. Differences in accessibility during study did not impact learning. In addition, Experiment 2 verified that the priming manipulation was successful: accessibility during the restudy phase was higher for words that were primed than for words that were not. Additionally, retrieval success was higher in the primed condition, but the results showed no impact of retrieval success or retrieval difficulty on learning.

It should be noted that accessibility was measured based on a specific memory test–a two-letter stem—and that this measure does not necessarily capture pure semantic activation. Thus, it is an open question whether a different measure of memory would result in differing levels of accessibility during the restudy phase. Similarly, during the test phase, it is possible that another type of memory test would have produced different results. Testing these possibilities is a direction for future research.

## 4. General Discussion

In two experiments, semantic priming was used to manipulate memory accessibility prior to restudy. The results showed that memory accessibility did not have a causal impact on learning, regardless of whether the restudy involved a presentation (Experiment 1) or retrieval with feedback (Experiment 2).

The correlation between lower memory accessibility and increased learning is well-established (e.g., Cepeda et al., 2006), but the cause of this correlation appears to be a third variable or variables. What might this third variable be? Perhaps certain processes that underlie forgetting cause decreased accessibility and increased learning, creating a correlation between these two variables. This possibility is consistent with the present findings, because the amount of forgetting prior to restudy was the same in the two conditions. It is also consistent with past literature; to my knowledge, in the previous research showing a correlation between learning and accessibility, the lower accessibility has always been due to increased forgetting.

To be clear, the present experiments cannot definitively support or refute the role of forgetting in learning because they did not examine the effect of forgetting. Nonetheless, multiple theories that explain the correlation between accessibility and learning are consistent with the idea of forgetting as the causal factor, such as theories implicating the benefits of contextual variability, inhibition, or interference (e.g., R. A. Bjork & Bjork, 2006; Cuddy & Jacoby, 1982; Estes, 1955; Storm et al., 2008). Whether forgetting plays a causal role in learning is a question for future research.

These findings suggest that retrieval difficulty, per se, should not be categorized as a desirable difficulty. Desirable difficulties are defined as conditions that decrease performance during study and increase learning (e.g., R. A. Bjork & Bjork, 2006; Soderstrom & Bjork, 2015). In the present experiments, the non-primed conditions decreased performance during study, but this manipulation had no impact on learning. This finding adds to a growing number of studies showing that retrieval success versus failure has little impact on learning (e.g., Kornell & Vaughn, 2016).

Along these lines, although success does not impact learning, making an attempt to think of an answer does (e.g., Knight et al., 2012; Kornell et al., 2009; Yan et al., 2014). It is possible that such attempts contributed to learning in Experiment 2, in both the primed and unprimed conditions. Investigating this possibility is a direction for future research. There are other directions for future research, such as examining the relative contributions of the semantic cue (the sentence) and the two-letter stem. Another question for future research is whether these results would hold under different experimental conditions, such as with other learning materials or longer retention intervals.

Relatedly, theories have been proposed that implicate difficulty as a cause of the spacing effect. According to these theories, studying something a second time can remind learners of the first study trial (e.g., Wahlheim et al., 2014) and induce a process of study-phase retrieval (e.g., Hintzman, 2010). The benefit of spacing occurs because retrieving a previously studied memory has more impact when doing so is more difficult (Benjamin & Tullis, 2010; R. A. Bjork, 1988; Pyc & Rawson, 2009). The present results do not call these theories into question, because these theories explain spacing effects and the present studies did not manipulate spacing. These findings do, however, specify that some ways of manipulating retrieval difficulty may lead to learning gains, but others—including manipulating priming—may not.

## 5. Concluding Comment

This research attempted to identify causal factors that influence learning. Several variables had no causal impact on learning, including retrieval effort, retrieval difficulty, and memory accessibility. While the literature shows a robust correlation between accessibility and learning, the causes underlying this relationship remain unknown. Future research could investigate causes that are rooted in processes that underlie forgetting to develop a deeper understanding of the causal factors that influence learning.

## Data Availability

The original contributions presented in the study are included in the article, further inquiries can be directed to the corresponding author.
